# Editorial: Women in multiple sclerosis and neuroimmunology: from bench to bedside

**DOI:** 10.3389/fneur.2025.1677618

**Published:** 2025-09-30

**Authors:** Jessy J. Alexander, Krista D. DiSano, Liliana Patrucco, Mireya Fernandez-Fournier

**Affiliations:** ^1^Department of Medicine, University at Buffalo, Buffalo, NY, United States; ^2^Department of Neurology, Geisel School of Medicine at Dartmouth, Hanover, NH, United States; ^3^White River Junction VA Healthcare System, White River Junction, VT, United States; ^4^Multiple Sclerosis Centre Buenos Aires Argentina (CemBA), Buenos Aires, Argentina; ^5^Neuroimmunology and Multiple Sclerosis Unit, Department of Neurology, Neurology and Cerebrovascular Disease Group, La Paz University Hospital–IdiPAZ, Madrid, Spain

**Keywords:** multiple sclerosis, neuroinflammation, complement, women, aging, immunosenescence

Multiple sclerosis (MS) is an inflammatory demyelinating disease with a striking female predominance. The ratio of women:men with MS in some countries has reached 4:1, a trend that has continued to rise over the past few decades (1). This burgeoning demographic reality highlights the need to understand the biological and clinical aspects of MS along with the impact of sex as a variable. This sex disparity, particularly evident in the relapsing/remitting form of MS, mirrors patterns seen in other autoimmune disorders, such as systemic lupus erythematosus, and reflects both biological and epidemiological shifts. The research featured in this special issue brings together the voices and work of women as both investigators and as subjects of inquiry. Their contributions are reshaping our understanding of MS and other neuroinflammatory conditions, extending from molecular mechanisms to clinical implications. This editorial highlights emerging insights in MS epidemiology, immunobiology, and therapeutic strategies while reinforcing the critical role of sex-specific research in neurology.

*A Shifting Epidemiological Landscape*: Between 1990 and 2021, the global incidence of MS rose by nearly 50%, with parallel increases in mortality and disability-adjusted life years (DALYs) (Wang et al.). Population growth, aging, and improved diagnostics such as the widespread availability of MRI and updated diagnostic criteria are suggested as major contributors to this rise. Developed countries report the highest MS burden, led by the United States in incident cases (1). The availability of disease-modifying therapies has transformed the course of the disease, reducing relapse rates and slowing progression. Environmental and lifestyle risk factors including Epstein-Barr virus infection, vitamin D deficiency, smoking and early life obesity, along with their interaction with genetics and disturbed homeostasis continue to shape the global prevalence of MS.

*Innate Immunity and Neuroinflammation:* Sex differences in MS extend beyond prevalence to fundamental immune mechanisms. A study by Laaksonen et al. revealed that positron emission tomography showed higher translocator protein (TSPO) binding in men with MS than in women with MS and even in healthy controls, reflecting activated microglia/macrophages and astrocytes. Their results suggest that central nervous system (CNS) cells may differ phenotypically and functionally between the sexes in MS and men may harbor a lower neuroinflammatory threshold that predisposes them to more aggressive neurodegeneration. Animal studies echo these findings, showing male microglia to be more pro-inflammatory, while female microglia may promote neuroprotection and repair (2). Estrogen likely contributes to this protective effect, although its precise role remains under investigation. Understanding these immunological dimorphisms is essential, especially as we move toward tailored interventions targeting cell function.

*The complement system*, an important arm of the innate immune system, is increasingly recognized as a central player in neurodegeneration. In their review, Negro-Demontel et al. explored the dual role of complement components in the brain: facilitating development and synaptic pruning under physiological conditions, while also driving pathology in diseases such as MS, Alzheimer's (AD), Parkinson's (PD), and Huntington's (HD). In MS, complement deposition (C1q, C3) and membrane attack complex (MAC) formation are found in active lesions, correlating with disease activity. In AD, complement overactivation has been implicated in synaptic loss. The review also introduces the concept of the intracellular complement “complosome” and its emerging relevance to CNS disease, in addition to the hypothesized link between persistent viral infections (e.g., EBV, HHV-6) and complement-mediated neurodegeneration. These insights point toward a future in which complement-targeted therapies could be tailored not only by disease but also by sex, age, and cell type.

*Aging and Immunosenescence:* While Disease-Modifying Therapies have revolutionized MS care, their safety and efficacy profiles in older adults remain underexplored. Sabin Muñoz et al. presented a compelling case of a 63-year-old woman with late-onset MS who developed a primary cytomegalovirus (CMV) infection while on dimethyl fumarate (DMF), despite having no documented lymphopenia. Immunosenescence, the natural decline in immune function with age, may alter both therapeutic responses and susceptibility to opportunistic infections, demanding a nuanced, individualized approach to treatment selection in older patients. This case highlights the critical need for age-inclusive research, as adverse events related to aging and immunosenescence are likely to be underreported in clinical trials that predominantly enroll younger individuals or enforce age-based enrollment restrictions.

*Biomarkers and the Challenge of Disease Subtyping:* Identifying reliable biomarkers for MS subtypes remains a critical frontier. Desu et al. reviewed the utility of cerebrospinal fluid (CSF) neurofilament light (NfL) levels across MS phenotypes. Although NfL serves as a reliable marker of axonal damage and correlates with disease activity, the authors demonstrated that NfL cannot clearly differentiate between the relapsing-remitting MS (RRMS) and progressive MS subtypes, restricting its value as a biomarker for disease progression. The field may benefit from integrating multiple biomarker modalities, including fluid biomarker panels (e.g., tissue injury and inflammatory markers), neuroimaging, and clinical indicators. Longitudinal, multi-modal assessments could yield more accurate predictors of progression and therapeutic response.

*Broader Implications of Sex in Neurodegeneration, Beyond MS*: Sex differences are not confined to MS. In Huntington's disease (HD), although genetic penetrance is equal across sexes, women often experience more severe progression and a heightened psychiatric burden, particularly depression. As Risby-Jones et al. report, female glial cells, including microglia, astrocytes, and oligodendrocytes, demonstrate heightened inflammatory responses and impaired repair capacity. These findings raise the possibility that glial-targeted therapies may require sex-specific calibration. For example, female astrocytes show higher GFAP expression and reduced phagocytic activity, while female oligodendrocytes may struggle with differentiation and remyelination. Therapeutic strategies that support glial resilience may therefore benefit from a tailored design based on sex-specific cellular responses.

*Looking Ahead: From Inclusivity to Personalized Precision*: This Research Topic of studies affirms that sex is a critical lens through which all neuroimmunology research must be examined. MS and other autoimmune diseases serve as compelling examples, given their female predominance, immunological complexity, and clinical heterogeneity, and provide an ideal model system for integrating sex-focused approaches into both research and care. This Research Topic also advocates for the critical need for diversity, not only in study populations, but also in scientific leadership. The strong presence of female scientists in this issue and their collective work embodies the translational ethos of bridging the gap from bench to bedside. Future research must therefore continue to champion integrative strategies that incorporate age, sex, immune profile, and neurobiology. Through such dedicated efforts, precision neurology can become more than a goal; it can become the standard.
